# SNMMI Clinical Trials Network Research Series for Technologists: Imaging Contract Research Organizations, Nuclear Medicine Technologists, and the Role They Play in Medical Imaging Research

**DOI:** 10.2967/jnmt.123.266111

**Published:** 2023-12

**Authors:** Matthew C. McMahon, Paul Galette

**Affiliations:** 1 Takeda Development Center Americas, Lexington, Massachusetts; and; 2 Telix Pharmaceuticals (US) Inc., Fishers, Indiana

**Keywords:** contract research organization, image acquisition guideline, charter, central read, query management, efficacy

## Abstract

Clinical imaging research is a fast-growing, complex, and integral part of drug and therapy discovery and development. Research sponsors rely on outside vendors to manage their trials and deliver results they hope will demonstrate the efficacy of their product. Specialized vendors known as imaging contract research organizations have teams of highly trained and specialized professionals who lend their expertise to all aspects of imaging research management, of which nuclear medicine technologists are key team members. This article is part of the Clinical Trials Network Research Series for Technologists and will help provide an overview of an imaging research study from initiation to data delivery and the roles that nuclear medicine technologists and other imaging professionals play.

Nuclear medicine technologists (NMTs) are highly trained, educated, and qualified health-care professionals, who are often credentialed in multiple imaging modalities. Attention to detail and adherence to strict rules and regulations are requirements for patient care, the handling of radioactive materials, and, ultimately, the administration of radiopharmaceuticals and, potentially, ionizing radiation for the acquisition of medical images or the treatment of patients. NMTs routinely follow specifically designed protocols to ensure high-quality patient care, ultimately generating quality images and data for interpretation by their departmental radiologists or nuclear medicine physicians. This professional skill set and experience, although required for clinical care, also makes NMTs potentially appealing to research and pharmaceutical companies outside clinical practice.

Ensuring that images are acquired in an accurate and technically appropriate manner is critical for patient care management. This becomes even more important when images are acquired as part of research trials, in which medical imaging can support endpoints related to drug or treatment efficacy, safety, or even the assessment of a novel imaging technique (e.g., a new diagnostic PET imaging agent or analysis method). Clinical trial images must be acquired uniformly across participating imaging centers and often longitudinally, as well as in accordance with the local rules and regulations of either the institutional review board or the radiation safety committee as pertains to research imaging. Therefore, the companies testing products, treatments, or therapies rely heavily on adherence to strict image acquisition and submission guidelines by the NMTs and other imaging technologists or professionals at the clinical center. Adherence to these guidelines ensures that images can be accurately interpreted and analyzed in support of varying disease criteria assessments and research endpoints. The future use of imaging, collected as part of a clinical trial, depends on this accuracy.

The success of clinical trials relies on accurate work by NMTs, but their value reaches beyond the clinic as well. Pharmaceutical companies, academic institutions, and contract research organizations (CROs) also seek out seasoned technologists to fill roles on their own imaging teams. NMTs can use their skills to assist in aspects of imaging trial design, study implementation, and analysis. These are roles that improve both the accuracy of the images and the validity of the data analyses. Experienced NMTs are often approached, or even recruited, to fill these job opportunities, thus bolstering the capabilities of the imaging teams at these outside companies. Such opportunities allow NMTs to further their personal careers and increase their involvement in the greater nuclear medicine community.

CROs are companies or entities that “provide sponsors (pharmaceutical, biotech and medical device companies) with research management services” (1*,*^2^). In this context, CROs are generally regarded as providing clinical services as would pertain to drug/pharmaceutical management, clinical monitoring requirements, site management, and other clinically related tasks and are bound to follow the good practice guidelines in title 21 of *Code of Federal Regulations,* part 11. Some of these entities may have individuals as part of their teams who have a grasp on imaging concepts. However, there are specialized CROs that focus only on imaging procedures, assessments, and analyses. Understandably, these companies are called imaging CROs (iCROs). iCROs have teams comprising radiologists, physicians, scientists, imaging technologists (including NMTs), and other imaging professionals. These iCROs cover every aspect of executing an imaging research study, including creation of critical imaging documents, image receipt and processing, management of central reads, data analysis, and delivery of results to the sponsors. NMTs can hold positions in each of these roles. Their input is valuable both for nuclear medicine–related studies or endpoints and across all studies that include medical imaging.

## INITIAL INTERACTIONS

iCRO interactions with potential sponsors typically begin with early engagement, including informal chance meetings or impromptu conversations. Large meetings or symposia, such as the Society of Nuclear Medicine and Molecular Imaging annual meeting, create the perfect opportunity for collaborations to spark. Critical support vendors (radiopharmacies, capital equipment companies, iCROs) leverage these meetings to present themselves to potential partners, hoping to meet and chat with study sponsors. These interactions are not one-sided, however, as sponsors often have projects or studies planned or conceptualized and seek out potential partners at these large conferences as well. At these meetings, capabilities are briefly discussed, contact information is exchanged, and future meetings are scheduled to elaborate further into the details of the exact support required.

These subsequent meetings are much more formal. A potential vendor will gather its team of experts to compile overall experience and general capabilities required to support the needs of a sponsor. NMTs are often critical members of these support teams, as they can speak directly to the complexities surrounding nuclear medicine imaging and other imaging modalities as well. NMTs can provide the details necessary to successfully establish relationships at clinical sites with the imaging teams, help to implement universal imaging protocols, and troubleshoot issues that may arise during image acquisition or data transfer. However, image management is only a portion of the support provided as part of study execution. Therefore, iCROs must be able to speak directly to their ability to provide full-service support of an imaging study from conception to study closure, which includes contract and budget negotiations, critical internal and site-facing document execution, data management, central read management, data delivery, regulatory adherence, and many other key aspects. These meetings and interactions are high-stakes situations, as the management of an imaging-based study can make or break the success of the sponsor’s study.

If a sponsor is satisfied with the overall presentation and wishes to engage an iCRO to support the study, the sponsor will present its study synopsis, or protocol, to the vendor and make a formal request for proposal. This request-for-proposal process allows the iCRO to tailor a specific solution to support the exact needs of the study given the study endpoints and objectives, imaging modalities included, central review criteria, and data management requirements. Like the capability presentation, these requests for proposal outline in exacting detail how a study will be run from contract execution through study closeout. In addition, a line-item budget will be prepared to document the price for executing each aspect of managing a successful study. Negotiations do occur in these initial engagements to ensure that the sponsors are paying fair market value for the support they will receive, based on industry standards. Once all aspects of contracting and oversight are aligned, the contracts are executed, and the study is turned over to the iCRO clinical operations and scientific teams to begin implementation.

## ICRO INITIAL ACTIVITIES

**FIGURE 1.**
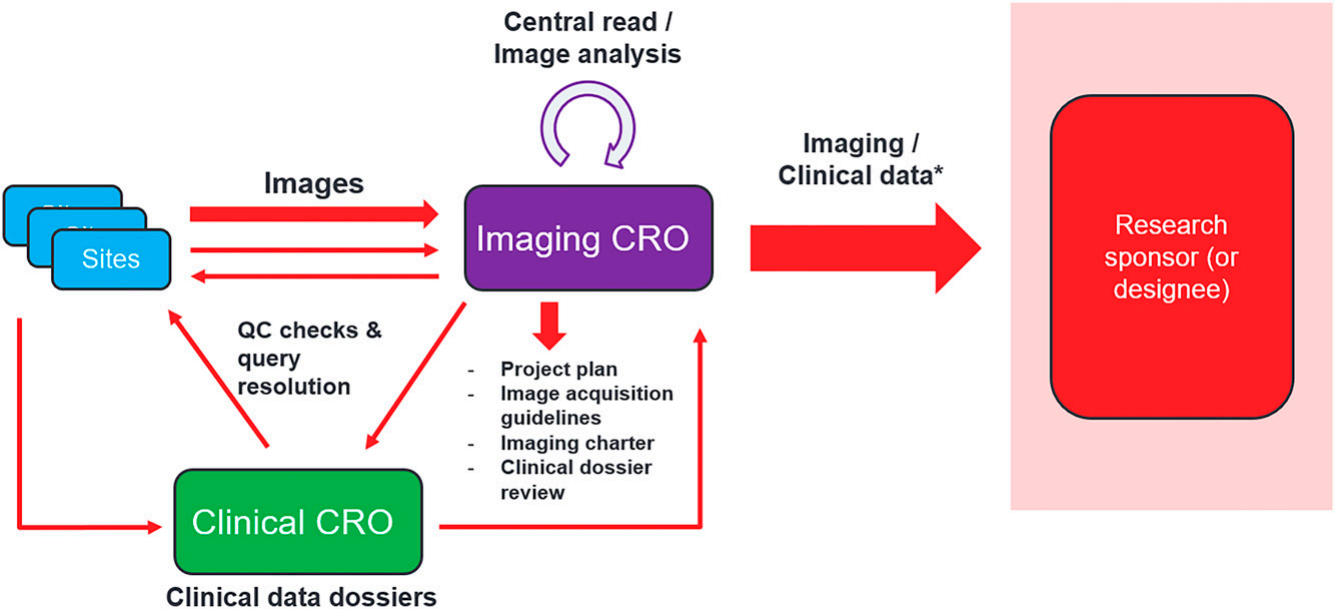
Graphic outlining life cycle of medical research image from image acquisition at site through data delivery to research sponsor. *Imaging and clinical data may require reconciliation efforts between iCRO, clinical trial sponsor, and clinical CRO when applicable. QC = quality control.

All aspects of study management, from an iCRO perspective (Fig. 1), require attention to detail and dedication. However, one group lends its expert experience and knowledge of the imaging sciences, which will hopefully better lead to the overall success of the study and, ultimately, the success of the sponsor’s aims. iCROs have various names for these groups—scientific affairs or scientific and medical services—but all provide highly specialized input. As mentioned previously, the endpoints, imaging modalities, and other specifics of the study will typically dictate who is assigned to each study. Scientific groups commonly consist of imaging technologists, radiologists, and academic researchers who are trained and have extensive experience in each of these specific aspects. Additionally, these iCRO team assignments can be scrutinized by the sponsor to ensure that the team is in alignment with the study and sponsor expectations. Potential substitutions or replacements can be made at the discretion of the sponsor.

One of the first steps in getting a project started, once it has been awarded, is to begin the study start-up phase. The iCROs prepare critical documents that outline study management and create an overarching communication plan. They are also responsible for drafting site-facing documents that prescribe the imaging procedures and outline submission details. If additional central analysis is desired, guidance documents are created. These include image review charters or image analysis plans that describe the precise way in which images will be assessed or analyzed. These documents will be authored in collaboration with the study sponsor and may be provided to regulatory authorities for review or approval. Because these documents outline the entire course of the study, it is imperative to get them in place before study work can truly begin.

An additional document that is generated during the start-up phase is the project plan. The project plan is designed to outline all the key aspects of the study and the methods and timelines for their completion. This document contains information from the study protocol, including the schedule of events and the study endpoints or objectives. All study service activities, along with the responsible parties, are outlined so that there are clear expectations on who will perform which task and how quickly results can be expected. In addition to these timelines, the key deliverables are outlined: central read results, image transfers, and the frequency with which these events occur. The project plan will serve as one of the main governance documents for the life of the study and keeps all involved parties accountable for their actions, including the iCRO, its team members, and the sponsors and other contracted vendors.

Once the project plan is complete, site-facing documents must be drafted. If images are to be collected as part of a clinical trial, they must be acquired in a uniform and precise manner across all imaging sites. Image acquisition guidelines or site manuals outline the role of the contracted clinical imaging center along with the responsibilities of each participating staff member. Some of the key aspects of these manuals are to provide contact information for the iCRO and sponsor, technical requirements, imaging frequency information, procedures for submitting images to the iCRO, and troubleshooting guides or query resolution plans. Each of these aspects is extremely important when the images are used for a central review or analysis. In addition to the routine study acquisition aspect, these manuals also outline potential site qualification steps and procedures required to ensure that the site is capable of successfully participating in the study and that the cameras or equipment used for imaging meet the standards required. The manuals may also describe the use of phantom images to ensure that detailed quantitative analyses can be performed successfully. Sites can be required to acquire specific phantoms, potentially on a repeat basis, so that iCROs and sponsors can test camera performance and ensure the overall quality of the images and equipment.

To ensure that sites fully understand the material and requirements outlined in the manuals, iCROs may also be contracted to train sites. This training can be either in person or, more likely, virtual via a web conference. The iCRO reviews the entire manual with the site representatives to ensure that the technologists and site staff understand any qualification procedures, the required imaging, the collection of clinical imaging data, and the expected turnaround time for uploading the images and data to the iCRO after acquisition. It is also important that the sites understand the image query process and how to resolve outstanding issues. Queries are generated by the iCRO when something is missing or potentially incorrect, including items as simple as clinical imaging data missing from the submission or as complex as improper application of a postprocessing filter. It is important for site coordinators and technologists to have a clear communication path with the iCROs so that any issues can be quickly resolved.

## BLINDED REVIEW

Depending on the trial needs, blinded independent central review can be implemented to ensure no bias during the assessment of medical imaging. This is commonly used when the primary or key secondary endpoints are based on measurements or specific review criteria. The central review charter, or site analysis plan, outlines the way that the clinical trial images will be analyzed or assessed. Depending on the disease being evaluated, the mechanism of action of the drug, the therapy, and the imaging modalities that are being leveraged to assess the disease, there are many ways in which a centralized read or analysis can be performed. iCROs are often responsible for the setup, conduct, and execution of the blinded independent central review paradigm. Rather than being produced as a guidance for site staff, like the image acquisition guideline document, this charter is created specifically to describe the analyses performed by a contracted central reader, often in a blinded setting. It is a robust document that contains a tremendous amount of information. This includes an introduction to the study as well as the key endpoints or objectives. Additionally, it outlines the roles and responsibilities for the members of the iCRO, the central readers themselves, and the sponsor. It defines the schedule of events as pertains to study images, the way the images will be assessed or analyzed, when and if any clinical data will be applied to central reads, central reader performance monitoring procedures, and the overall management of the read process. Once the charter document is finalized, separate technical teams configure or develop a trial-specific review application to collect the study-specific assessments during the blinded reads. Depending on the complexity of the analysis techniques or criteria used, this review application may include the drafting of separate technical documents distilling charter requirements before programming, testing, and validation.

Part of the central read may include the integration of clinical data that must be delivered and provided to the iCRO via the partnered clinical CRO. Working with the scientific team at the iCROs, clinical CROs will prepare clinical dossiers that capture clinical data points. Some of this information may include prior radiotherapies, prior therapies, blood work or tumor marker values, or biopsy results. It is the responsibility of the scientific team at the iCRO to ensure that whatever data are required to allow the central readers to provide a full and complete criteria-based assessment has been captured at the site level and will be delivered to the iCRO for the central readers.

This process can be quite complex, which is why all the central readers must be fully trained and qualified on the review charter contents before beginning any trial-specific reads. It is the responsibility of the scientific group at the iCRO to conduct this training. As part of this training, unique central review criteria rules will be presented, along with representative images or cases, which allow the reader to attempt to apply the rules in a training environment. Some of the criteria that are supported by central reviews may include RECIST (3), immune RECIST (4), Response Assessment in Neuro-Oncology (5), Lugano (6), or Prostate Cancer Clinical Trials Working Group 3 (7), depending on what disease process is being assessed. It is critical that the readers understand their responsibilities and timelines for delivery before beginning work on actual live study images.

Once the study moves into the central read or study execution phase, it is also the responsibility of the scientific group to continuously monitor the performance of the central readers against the rules of the imaging charter and for correct application of the prescribed imaging criteria. As part of the central read process, readers leverage the previously mentioned review application to record measurements and response assessments that tabulate the data and create an electronic case report form. These forms are engineered to perform in accordance with all the rules and contain various edit checks. They are also designed and coded to prevent central readers from making errors that would go against the charter or criteria rules. However, readers are allowed to use and apply their expert judgment when necessary, thus adding to the complexity in creating these read applications.

To ensure that the central read data delivered to the sponsor are accurate and that central reads have been performed in accordance both with the rules of the imaging charter and with the review criteria, the scientific group often creates quality control checks that are run against the electronic case report forms completed by the readers. These checks review all radiologic judgments, measurements, or response assessments (both time point and global/overall) and ensure alignment with the guidelines. Should any errors occur, corrective actions may be required. Some of these actions include training point reminders to the reviewers, which outline specific rules or parts of the charter that may have been forgotten. If the mistakes are severe enough and there is a direct effect on downstream reviews, then the time point at which the mistake was made may need to be rolled back, meaning that all the central read data are erased and the readers are asked to repeat the assessments, with strict reminders of how reads are meant to be performed according to the charter or review criteria. It is the hope that the central readers make few and minimal mistakes, but these means of correction are in place to ensure the quality of the data. Any changes or corrections are fully auditable and are shared with the sponsor.

The scientific team not only monitors a reviewer’s initial performance on central reads but also may assess reviewer variability or performance via a secondary read review. This type of review happens when the reader may reread previous subjects to ensure they are consistent not only with the reader’s own previous assessments but also with other readers’ assessments. Like standard quality control of central reads, if mistakes are being made that require broader correction, actions such as training point reminders can be taken. If major action is necessary, all readers can be retrained to bring them back to consensus.

## IMAGE PROCESSING

The central read aspect of an imaging-based research trial may be the crux of an iCRO’s responsibility, but central reads of images would not be possible without a group within the iCRO organization known as image operations, which manages receipt and preparation of images. This group works closely with the scientific group to create an outline of acceptable image and acquisition parameters that meet the requirements of the schedule of events, the imaging charter, and image assessment criteria. When drafting the image acquisition guideline, the scientific group prescribes uniform image acquisition parameters, which are often found in and defined by practice standards. These standards may come from societal, or practice, guidelines as published by the Society of Nuclear Medicine and Molecular Imaging or European Association of Nuclear Medicine or as part of peer-reviewed methodology research papers. It is the expectation, then, that sites acquire images exactly as prescribed in the image acquisition guidelines. However, sites are not always able to meet these expectations because of standard-of-care limitations, local rules or regulations applying to radiation exposure, or, in some instances, simply mistakes that are made. It then becomes the responsibility of the image operations team to ask the sites questions to clarify and potentially rectify these issues. Query resolution may require that new images or missing clinical information be submitted or, in extremely rare situations, entirely new image sets be acquired.

Image operations also ensures that all received images are properly formatted to protect both the integrity of potentially biasing information and subject confidentiality by performing detailed checks of the pixel data and Digital Imaging and Communications in Medicine (DICOM) metadata fields. As images come in from the sites, quality control procedures ensure that all images are scrubbed of identifying information (site name, subject demographics, etc.) and meet all technical requirements. Image operations also ensures that image acquisition dates match the windows of acceptance around prescribed imaging visits.

The image operations team can also be responsible for preliminary analyses. These operations teams contain professionals from across the imaging spectrum, often including former imaging technologists, all of whom have extensive knowledge on and experience with analyzing images as part of their responsibilities in the clinic. There are some contracted quantitative image analyses studies that require regions of interest to be drawn or created. In some settings, it may be efficient and cost-effective to have the image operations team perform its initial analysis and then pass it on to either the central reader or an internal scientist for review, revision, and approval. Because central reader time is expensive, it may be cost-prohibitive to ask the central reader to perform all analyses individually.

## OPERATIONS/PROJECT MANAGEMENT

Another team within the iCRO framework is the operations team. This team contains individuals with a wide range of professional backgrounds, including technologists. Operations teams are commonly known as project managers and are responsible for all aspects of study management. Much like the scientific team, the project manager is assigned to the study as soon as the contract is signed. The project manager is responsible for preparing all the materials to conduct an initial study kickoff meeting, which is hosted by the iCRO and includes members of the iCRO’s scientific team, the iCRO’s portfolio managers for the sponsor, key sponsor representatives, and other individuals from other contracted entities who may interact or need the data produced by the iCRO. In this meeting, the operations team will outline key timelines and the next steps to get the study started, define milestones for delivery, and provide effective communication channels for all team members.

Once the kickoff meeting is complete and study start-up tasks begin, the operations team ensures that the study remains on the critical path and meets all deadlines. All documents need to be reviewed and executed by all responsible parties, critical training sessions for clinical sites and readers must be scheduled and completed, and all documentation must be completed and filed before central read initiation. Once the reads begin, the operations team ensures that images are processed and prepared by the image operations team and then assigns time points to the central readers. In addition, if clinical dossiers are needed to support specific criteria assessments, the operations team ensures that the dossiers are applied to the correct subject and the proper time point. The operations team also ensures that reads are completed according to the prescribed timelines, often 5–10 business days. If follow-up with the reviewers is necessary, the operations team reaches out to the reviewer and engages the reviewer operations team for escalation. The reviewer operations group is a separate entity within the iCRO responsible for managing the external central reviewers. This group handles all correspondence with the reviewers as well as their contracting, performance, and potential remdediation plans when required.

The project manager is also responsible for overseeing the image query process and ensuring a timely response from the clinical sites. This oversight ensures that the site fully completes all action items, resulting in closure of each query. Similarly, the operations team, working closely with the scientific team, ensures that training point reminders are received by the central reviewers and that any rollbacks of central reads are performed when corrective actions are necessary. Once all reads are complete, the operations team coordinates activities with the data management teams to ensure timely compilation of the data and that any required exports of the cleaned data are delivered to the sponsor according to critical timelines.

Ultimately, the operations team serves as the primary point of contact between a study sponsor and the contracted iCRO. The operations team needs to maintain a relationship with each critical team member to ensure that the study runs as smoothly as possible, that timelines are met, that accurate data are delivered according to the contracts, and that all questions or concerns from the sponsor and its delegates are addressed.

## CONCLUSION

For research studies with an imaging component, iCROs have a role critically important to the sponsor. The sponsor relies heavily on the combined experience of the teams at iCROs as pertains to running a study in general and to having precise knowledge and experience working with specific imaging modalities or central review criteria. It is hoped that the reader of this article will now have a better understanding of the iCRO’s role in supporting the development pathway of a potential therapy and that NMTs will have a critical role in that success.

## DISCLOSURE

Matthew McMahon works for Takeda Development Center, Americas, and owns future stock options in the company. No other potential conflict of interest relevant to this article was reported.
